# An Update on Molecular Diagnostics for COVID-19

**DOI:** 10.3389/fcimb.2020.560616

**Published:** 2020-11-10

**Authors:** Khursheed Ul Islam, Jawed Iqbal

**Affiliations:** Multidisciplinary Center for Advanced Research and Studies, Jamia Millia Islamia, New Delhi, India

**Keywords:** Severe Acute Respiratory Syndrome Coronavirus 2, COVID-19, diagnostics, reverse transcription-PCR, SHERLOCK, CRISPR-Cas12a

## Abstract

A novel strain of Severe Acute Respiratory Syndrome Coronavirus 2 (SARS-CoV-2) disease (COVID-19) has been recently identified as an infectious disease affecting the respiratory system of humans. This disease is caused by SARS-CoV-2 that was identified in Chinese patients having severe pneumonia and flu-like symptoms. COVID-19 is a contagious disease that spreads rapidly *via* droplet particles arising through sneezing and coughing action of an infected person. The reports of asymptomatic carriers changed the scenario of symptom based-diagnosis in COVID-19 and intensified the need for proper diagnosis of the majority of the population to combat the rapid transmission of virus. The diagnosis of positive cases is necessary to ensure prompt care to affected people and also to curb further spread of infection in the population. Collecting samples at the right time and from the exact anatomical site is crucial for proper molecular diagnosis. After the complete genome sequence was available, China formulated RT-PCR as a primary diagnostic procedure for detecting SARS-CoV-2. Many in-house and commercial diagnostic kits have been developed or are under development that have a potential to lower the burden of diagnosis on the primary diagnostic techniques like RT-PCR. Serological based diagnosis is another broad category of testing that can detect different serum antibodies like IgG, IgM, and IgA in an infected patient. PCR-based diagnostic procedures that are commonly used for pathogen detection need sophisticated machines and assistance of a technical expert. Despite their reliable accuracy, they are not cost-effective tests, which a common man can afford, so it becomes imperative to look for other diagnostic approaches, which could be cost effective, rapid, and sensitive with consistent accuracy. To make such diagnostics available to the common man, many techniques can be exploited among, which are Point of Care (POC), also known as bed side testing, which is developing as a portable and promising tool in pathogen diagnosis. Other lateral flow assay (LFA)-based techniques like SHERLOCK, CRISPR-Cas12a (AIOD-CRISPR), and FNCAS9 editor-limited uniform detection assay (FELUDA), etc. have shown promising results in rapid detection of pathogens. Diagnosis holds a critical importance in the pandemic situation when there is no potential drug for the pathogen available in the market. This review sums up the different diagnostic approaches designed or proposed to combat the crisis of widespread diagnosis due to the sudden outbreak of a novel pathogen, SARS-CoV-2 in 2019.

## Introduction

Diagnosis is a major aspect in tackling the consequences of any deadly contagious diseases. Diagnostic tests demonstrate the presence or absence of an infectious agent. Early and better diagnosis has helped in limiting fatalities due to highly infectious and contagious diseases in the past (Caliendo et al., 2013). Diagnosis has an empirical role in such diseases that are caused by any novel pathogen for which the population is not pre-immune. COVID-19 is one of such infectious diseases, highly contagious and deadly (Cascella et al., 2020). COVID-19 was found to be caused by a novel viral pathogen named as SARS-CoV-2. COVID-19 originated in Wuhan city of China and spread to almost the entire world (Rothan and Byrareddy, 2020). In January 2020, China reported the outbreak of SARS-CoV-2 that was further declared as pandemic owing to indiscriminate and rapid spread to different parts of world. Among the first 41 cases of COVID-19 reported by Huang et al., most of the patients had a history of exposure to Wuhan wild animal market. Patients had several symptoms like cough, fever, dyspnea, fatigue and radiographic indication of pneumonia (Huang et al., 2020). Whole genome sequencing of SARS-CoV-2 led scientists to design testing protocols to detect the pathogen in the affected people and also provided an insight in the phylogenetic study of the virus. It was elucidated that SARS-CoV-2 belongs to the family of beta-coronavirus, which include SARS-CoV and Middle East Respiratory Syndrome (MERS) viruses (Zhou et al., 2020a). The origin of this virus is still a matter of debate; however, bats are thought to be its primary source. SARS-CoV-2 was identified on 3^rd^ January 2020 in the bronchioalveolar lavage fluid samples taken from a patient in Wuhan, China (Zhu et al., 2020). As the understanding regarding COVID-19 disease evolved, it was observed that SARS-CoV-2 infection spreads *via* asymptomatic carriers (Rothe et al., 2020), which further prompted health workers to increase the diagnostic frequency among the masses.

As evident from the previous outbreaks of SARS-CoV in 2003 and MERS in 2012 (Cheng et al., 2007), a sensitive, specific and rapid diagnosis for COVID-19 are crucial in identifying positive cases, tracing its contacts, finding the source of virus and finally rationalizing the measure of controlling infection. During the early period of the epidemic, complete sequencing of SARS-CoV-2 facilitated the specific primer-designing and laboratory diagnosis of COVID-19 (Lu et al., 2020). On 23^rd^ January 2020 the first protocol of RT-PCR for COVID-19 diagnosis was published. This assay targeted viral genes related to Nucleocapsid (N), RNA-dependent RNA polymerase (RdRp), and Envelope (E) protein. RdRp was found to be most sensitive among these variants. This assay was using two probes; one probe called ‘Pan Sarbeco- probe’ detects SARS-CoV-2, bat-related SARS coronaviruses and other coronaviruses. However, another probe called RdRp-p2 was specific to SARS-CoV-2 only (Chan et al., 2020). Jasper Fuk-woo Chen et al. had developed a novel RT-PCR based diagnostic method for COVID-19 called RdRp/Hel, which was found to be more sensitive and specific than the previously published RdRp-p2 method (Chan et al., 2020) RT-PCR is a highly specific and sensitive diagnostic method for infectious disease detection like COVID-19. As this method is based on the detection of nucleic acids, it enables an early diagnosis of COVID-19 by detecting SARS-CoV-2 RNA in the patient sample (Corman et al., 2020). Despite being specific, sensitive and rapid, PCR-based methods lie restricted to a clinical laboratory with sophisticated equipment and trained personnel that limits its use as a Point of care diagnosis (Notomi, 2000). There is a dire need of alternative diagnostic techniques to use as point of care tests in the current pandemic. A dramatic burden on public health and society due to sudden outbreak of SARS-CoV-2 virus limited the use of sequencing and RT-PCR as a tool of rapid diagnosis because both these techniques take much time to generate test result and are affordable to a very limited section of population, thus making it imperative to develop new point of care diagnostic techniques that can compete with RT-PCR in terms of sensitivity and are easily affordable to common man. Many techniques can be exploited to make rapid and affordable testing available to common people that include Point of Care (POC) also known as bed side testing. POC testing is developing as a portable and promising tool in pathogen diagnosis (Yeh et al., 2017), a major example is the discovery of CRISPR COVID that has been shown to deliver comparable specificity and sensitivity as that of RT-PCR and sequencing based meta genomic diagnostic approaches (Hou et al., 2020). Moreover, lateral flow assay (LFA) is one of the latest POC techniques, which works on the principle of fluid flow, and it is employed in immune assays targeting protein, DNA or RNA based samples (Tang et al., 2017). According to a study conducted by Banerjee et al. in 2018, these POC based techniques can be utilized for viral detection in patient samples (Banerjee and Jaiswal, 2018). Other LFA-based techniques like SHERLOCK (Specific High Sensitivity Enzymatic Reporter unLOCKing) developed by Feng Zheng’s lab is aiming to be a fast, cost-effective and sensitive technique for pathogen detection (Gootenberg et al., 2018). All-In-One Dual CRISPR-Cas12a (AIOD-CRISPR) is another promising technique known to be fast, sensitive and a very efficient system for pathogen detection (Ding et al., 2020). These techniques can be employed to lower the burden of costly diagnostic procedures as well as lowering time consumption during any pandemic disease outbreak. This review will elaborate the diagnostic procedures, which are currently in use and will also highlight other new techniques that can be utilized for rapid and cost-effective diagnosis of COVID-19.

## Sample Collection and Biosafety Measures

High level of viral loads in the upper and lower respiratory tracts have been demonstrated in COVID-19 patients within 5-6 days of the onset of symptoms (Pan et al., 2020). For an early diagnosis of COVID-19, nasopharyngeal or oropharyngeal swabs are recommended (Chan et al., 2004) (Zou et al., 2020), however, a single nasopharyngeal swab is a method of choice for health practitioners because patients can easily tolerate it and is safe for handling. To obtain a proper nasopharyngeal swab specimen, the swab must go deep into the nasal cavity eliciting tears in the patient (Druce et al., 2012). Collected swabs should be immediately transported using transport media to the diagnostic laboratory, ideally in refrigerated conditions (Druce et al., 2012). Patients with severe COVID-19 pneumonia have shown high viral loads in bronchoalveolar lavages, however, nasopharyngeal swabs were not compared in the particular study (Wang et al., 2020). These patients have also shown high viral RNA in fecal samples as well (Zhang W. et al., 2020). Thus the preferred method of collecting samples from advanced COVID-19 patients is from the stool or the rectal swabs (Cheng et al., 2004). For the safety of health practitioners and the proper processing of samples, it becomes imperative to take utmost precautions while collecting, transporting and processing the COVID-19 samples. In response to this, the health practitioners must use goggles, N95 respirators, gloves, full sleeve gowns or PPE kits in order to minimize direct contact with the COVID-19 positive patients (Karthik et al., 2020).

**Figure 1:**
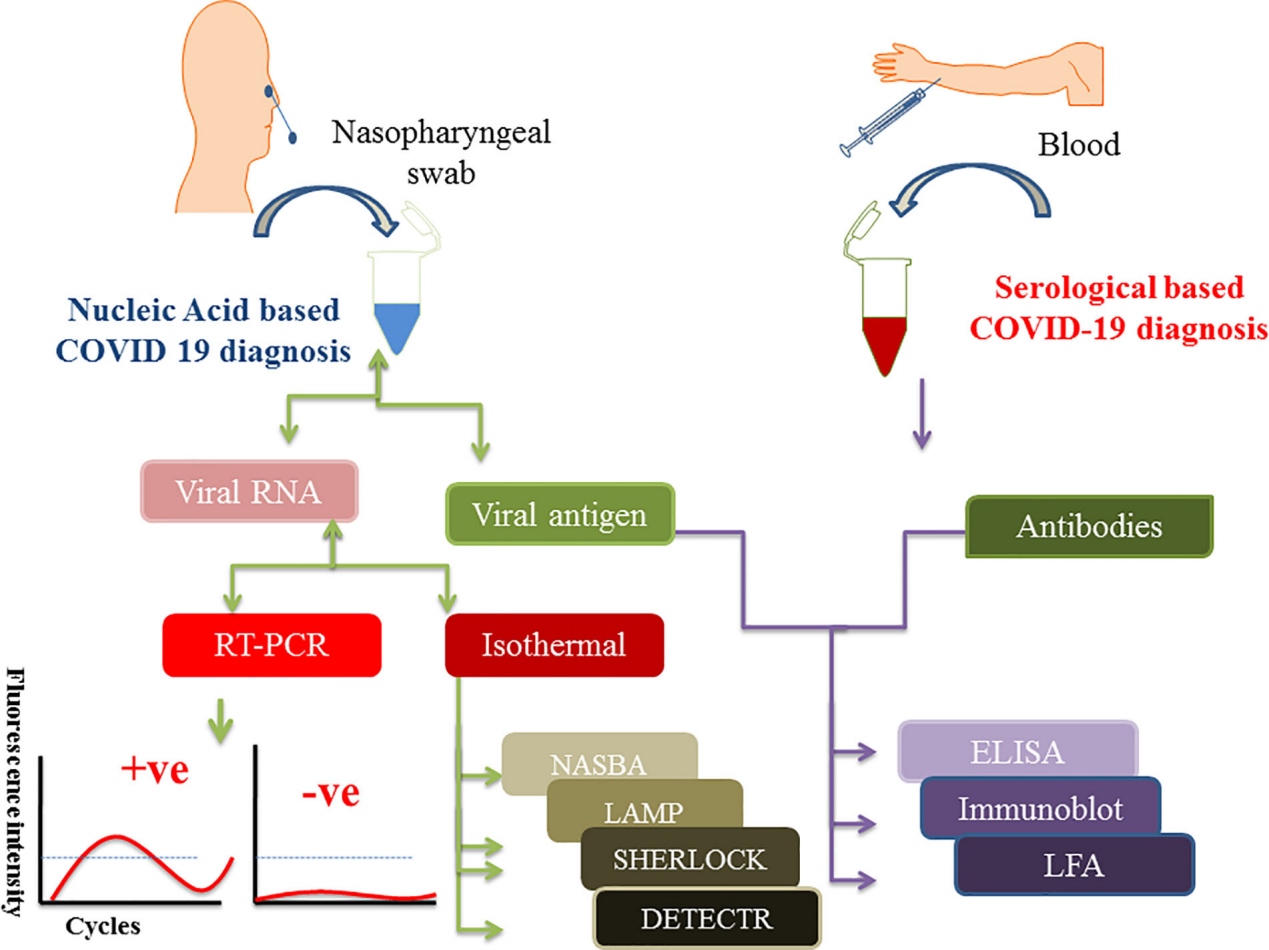
Figure depicts the scheme of sample collection and mentions different diagnostic approaches to detect SARS-CoV-2 infection. Nasopharyngeal swabs and serum samples are drawn from the patients which are further subjected to either nucleic acid or protein-based diagnosis depending on the need. Isolated viral RNA can be used for PCR, isothermal and CRISPR based methods whereas viral antigen and antibodies (IgG/IgM) are used for protein-based diagnosis like ELISA and point of care LFA methods.

During the early time of COVID-19 pandemic, a simple and convenient approach of collecting patient sample was a dare need to replace the painful nasopharyngeal swab collection process. In that scenario, Rutgers Clinical Genomics Laboratory came up with an idea of developing an RT-PCR based technique, which can detect SARS-CoV-2 RNA in self collected saliva samples. They developed an assay kit, which was commercialized as TaqPath™ COVID-19 combo kit. This strategy lowered the risk of contracting infection during sample collection by the health practitioners (Afzal, 2020). Sample collection for protein-based diagnosis like IgG/IgM and LFA, requires patients’ blood samples. **Figure 1** shows the schematics of specimen/sample collection for COVID-19 diagnosis as well as various nucleic acid and protein-based diagnostics approach.

## RT-PCR as a Frontline Diagnostic Method for COVID-19 Diagnosis

Molecular diagnostic approaches are appropriate as compared to other syndromic testing approaches because molecular diagnosis targets the genome or proteome of the pathogen thus making it a specific and reliable method of diagnosis (Zhou et al., 2020b). For a novel pathogen sequencing and diagnosis becomes imperative to recognize the nature of the pathogen and its genomic composition. Random amplification and deep sequencing strategies played a critical role in early identification of the SARS-CoV-2, which was further confirmed to be a member of the coronavirus family *via* different bioinformatics approaches (Briese et al., 2014). Using metagenomic sequencing, the first genomic sequencing was conducted for SARS-CoV-2 (Miller et al., 2019; Sheridan 2020a). On 10^th^ January 2020, the findings were made public and the sequences submitted to the sequence repository of GenBank (Wu et al., 2020). Release of whole genome sequence of SARS-CoV-2 to public databases made it easy for scientists to design primers and probes for conducting laboratory diagnosis of COVID-19 (Corman et al., 2020). After the identification of this virus, WHO recommended real time reverse transcription polymerase chain reaction (real time RT-PCR), which is a nucleic acid-based technique, as the frontline diagnostic approach to detect SARS-CoV-2 infection in suspected patients. RT-PCR is highly sensitive and can detect infection at minute levels of pathogen present in the patient sample. It is a nucleic acid-based technique used to amplify a target gene/nucleotide present in a sample, which helps in detecting a specific pathogen and discriminating it from other related pathogens. There are usually two possible ways of performing RT-PCR including one-step assay or two-step assay. One step assay consolidates reverse transcription and PCR amplification in a single tube thus making the process of detection rapid and reproducible; however, this assay provides a lower target amplicon generation. In case of two-step assay the reactions are carried out sequentially in two separate tubes making it time-consuming, but a sensitive assay compared to the one-step assay format (Wong and Medrano, 2005).

Although eleven nucleic acid-based protocols and eight antibody detection kits have been approved by the National Medical Product Administration (NMPA) in China, PCR was considered as a preferred diagnostic technique. The US Centers for Disease Control and Prevention (CDC) uses a one-step PCR format to diagnose COVID-19 (https://www.fda.gov/media/134922/download). The assay is carried out by isolating RNA from the sample and adding to the master mix containing forward and reverse primers, nuclease-free water, reaction mixture (reverse transcriptase, polymerase, nucleotides, magnesium and other additives). A PCR thermocycler is loaded with extracted RNA and mastermix, and the temperature is set to run the PCR reaction (https://www.fda.gov/media/134922/download). Cleavage of a fluorophore quencher probe during this reaction generates a fluorescence signal that is detected by the thermocycler, and the progress of amplification is recorded. Positive and negative controls must be included whenever running any RT-PCR reaction, which makes the interpretation of results easy and stringent (Chan et al., 2020). RT-PCR and some biosensor based diagnostic kits can detect SARS-CoV-2 nucleotides in fecal samples or sewage water that can be a warning of an infectious disease outbreak in the particular area. SARS-CoV-2 can survive from hours to days in the untreated sewage water (Orive et al., 2020).

RT-PCR is a sensitive and rapid detection tool in molecular diagnostics. It can detect and amplify even a few copies of specific genomic sequence in a variety of samples, but it depends upon certain aspects to deliver reliable results like proper collection, transport, storage, and processing of samples (Afzal, 2020). It has been used for detection of diverse viruses like Adenovirus, Rotavirus, Astroviruses and many enteric viruses isolated from fecal samples (Kowada et al., 2018). A Major drawback of this technique is the need for a well-equipped laboratory and technical personnel for handling the experiment, which cannot mitigate the increased demand of rapid testing during pandemic situations like COVID-19 (Bustin and Nolan, 2004). The RT-PCR based kits are highly expensive and take much time to deliver results thus making it essential to look for other rapid and reliable diagnostic methods (Hofman et al., 2020; Sheridan 2020b).

## Other Nucleic Acid-Based Diagnostic Techniques

### Nucleic Acid Sequence-Based Amplification (NASBA)

NASBA is an *in vitro* amplification process conducted in isothermal conditions. It is a two-step amplification process where the first step is the denaturation and the second step is a polymerase dependent amplification conducted isothermally (Compton, 1991). Fluorochromes are also added to the reaction in order to make it a real time-based observation. This technique has been further modified as a multiplex process called multiplex real time nucleic acid sequence based amplification (RT-NASBA), which can help in the concurrent detection of different viral infections (Mo et al., 2015). RT-NASBA has been proven to be 10–100 times more sensitive than Multiplex RT- PCR, owing to the isothermal conditions where no time is consumed in heating and cooling and production of copies is faster than RT-PCR. RT-NASBA has been previously used for detection of SARS-CoVs infections, and their sensitivity and specificity was seen to be parallel with RT-PCR diagnosis (Keightley et al., 2005). This technique can be a choice for the rapid diagnosis of COVID-19 during the current pandemic.

### Loop Mediated Isothermal Amplification (LAMP)

LAMP is a diagnostic technique that is comparatively less expensive, much sensitive and rapid than RT-PCR. This technique involves the selective amplification of target nucleic acids at a constant temperature, usually 60°C. In this technique 4 to 6 specifically designed primers are used to detect distinct nucleic acid sequences, moreover there is no requirement of initial template denaturation and reaction time is minimized up to 30 minutes using strand-displacement polymerases (Kashir and Yaqinuddin, 2020). For a colorimetric based analysis LAMP reaction mixture is added with hydroxynepthol blue (HNB) prior to amplification, thus avoiding cross contamination in the future (Malik et al., 2019). Lin Yu et al. have used another approach that combines reverse transcription with LAMP diagnostic technique (RT-LAMP) allowing direct detection of SARS-CoV-2 RNA. This technique was further coupled with a pH indicator, which helped in visual readout of the amplification reaction *via* color change in the reaction mixture (Yu et al., 2020). RT-LAMP based assay that targets S gene of SARS-CoV-2 developed by Hu et al., which showed 88.89% sensitivity and high consistency compared with RT-PCR based diagnostic methods. Time required for this assay was around an hour, considerably lower than RT-PCR (Hu et al., 2020). LAMP technique avoids the use of costly reagents and instruments, thus helping in reducing the cost of diagnosis with rapid results (Cascella et al., 2020). Various studies have highlighted the application of LAMP technique in detecting coronavirus infections in patient samples (Poon et al., 2004; Pyrc et al., 2011). It was further observed that 9 to 10 copies of viral RNA per reaction were sufficient to detect infection giving a 100 fold higher sensitivity than RT-PCR (Mori et al., 2001; Thai et al., 2004; Njiru, 2012). Many other reports are also available on the use of LAMP as a diagnostic tool which includes the work carried out by El-Tholoth et al. (2020) where they demonstrated a two stage LAMP design called COVID-19 Penn-RAMP strategy. This method can be carried out in closed tubes with either colorimetric or fluorescence detection. Their results are not only comparable to RT-PCR but also show 10 fold higher sensitivity when using purified targets (Kashir and Yaqinuddin, 2020). Penn-RAMP is based on the preliminary reaction where outer LAMP primers bind to amplify all targets concurrently through recombinase polymerase. After the first step, a second highly specific reaction initiated. The first reaction specifically uses outer LAMP primers, while the second reaction combines four other RAMP primers. This enhances the sensitivity of this method compared to normal LAMP (Song et al., 2017). For an improved diagnosis, Song’s group developed a two stage isothermal Penn-RAMP double stranded DNA amplification assay that combine LAMP and Recombinase Polymerase Amplification (RPA) in a single tube thus making the assay simple and less time consuming, further this method has been integrated to a smart phone application to make it highly accessible and point of care technique (Yang et al., 2020). The challenge related to the LAMP method is the primer optimization and reaction conditions.

### Point of Care Testing and COVID-19

Diagnosis of infectious diseases at the bed side of a patient where there is no need of sending patient samples to sophisticated labs is called as Point of Care testing (POC). POC has a very important role to play during community contagious infections because it enables communities to diagnose infection without the complex laboratory infrastructure. POC testing is the only option for the remote areas of any community. One of the Point-of-Care testing protocols is the LFA. Xiang J.; Yan M. et al. had used lateral flow antigen assay for COVID-19 diagnosis (Xiang et al., 2020). LFA is carried out on a strip which is a paper like membrane. The membrane has two lines coated on it among which one line contains gold nanoparticle-antibody conjugate and the other line contains a capture antibody. Patient samples are deposited on the strip. The strip draws proteins from the sample *via* capillary action. As the sample runs over the first line of the strip, antigen binds the nanoparticle-antibody conjugate. This complex then moves to the second line where the capture antibody immobilizes the complex which makes a red or blue line visible on the strip. Individual gold nanoparticles on the strip are red, however clustered gold nanoparticles in solution show a blue color. For IgM, LFA has shown 57% clinical sensitivity, 69% accuracy, and 100% specificity however LFA has shown 81% sensitivity, 86% accuracy, and 100% specificity for IgG. Clinical sensitivity equal to 82% was observed in the tests that detected both IgG and IgM (Xiang et al., 2020). These methods have comparatively less sensitivity than PCR and its variants. Researchers have tried to enhance the sensitivity of LFA-based methods by combining it with RT-LAMP and such related techniques. This combination has been used in MERS-CoV detection (Huang et al., 2018). Based on the principle of LFA, some techniques are specifically developed to detect viral pathogens. Specific High Sensitivity Enzymatic Reporter Unlocking (SHERLOCK) is one such technique developed by Feng Zheng’s lab. This technique is very cost-effective and rapid in diagnosing viral pathogens. It has been used for detecting Zika and Dengue virus in patient samples (Gootenberg et al., 2017). For cold-chain and long-term storage SHERLOCK reagents are subjected to lyophilization. SHERLOCK can detect at least 200 copies/ml of serum/urine for Zika viral RNA (Gootenberg et al., 2017). A protocol has been developed by Zhang et al. for diagnosis of COVID-19 using SHERLOCK. It is a three-step diagnostic process. Isothermal amplification, detection and visual readout are the three steps of diagnosis which take less than an hour for the final results (Zhang F. et al., 2020).

## CRISPR-Based Point of Care Diagnosis

CRISPR (Clustered Regularly Interspaced Short Palindromic Repeats) has emerged as a game changer in the molecular biology experiments. It has also reshaped the diagnostics of the current time. CRISPR are the DNA sequences found in bacteria and archaea that have been extensively used in gene editing experiments. They play an important role in antiviral defense as the sequences are derived from bacteriophages which have previously infected bacteria (Barrangou, 2015). Lots of CRISPR based techniques are currently in use or have a potential to be an option of point of care testing in pathogen diagnosis like SARS-CoV-2. All-In-One Dual CRISPR Cas12a, also termed AIOD-CRISPR is believed to be an ultrasensitive and accurate visual diagnostic technique. In order to initiate a highly specific CRISPR- based detection of nucleic acids a dual crRNA (CRISPR-RNA) is introduced in the reaction mixture (Jeon et al., 2018). All the components for nucleic acid amplification and CRISPR-based detection are mixed in a single tube and are subjected to isothermal incubation (37°C). This limits contamination because no separate pre-amplification and amplification is needed. AIOD-CRISPR assay has been engineered for detecting SARS-CoV-2 pathogen (Jeon et al., 2018). AIOD-CRISPR system uses a pair of Cas 12a- crRNA complexes generated by two distinct crRNAs. These complexes bind to corresponding sites closer to the primer recognition sites in the target sequence. The reaction takes place in a single tube that contains: Cas12a-crRNA complex generated separately, recombinase polymerase amplification (RPA) primers, recombinase, ssDNA-FQ reporters, strand displacement DNA polymerase, ssDNA binding protein and target sequences. Binding of Cas12a-crRNA complex to the target sequence leads to the cleavage of nearby ssDNA-FQ *via* activated Cas 12a this process produces a fluorescence signal (Ding et al., 2020). In the study carried out by Xiong Ding et al. a plasmid containing 384 nucleotide N gene cDNA was used as the target to develop AIOD-CRISPR assay. This assay could detect 1.3 copies of SARS-CoV-2 N plasmid in both visual and real time detection within 40 minutes. AIOD-CRISPR has the benefit of being used as a Point of Care diagnosis for pathogenic diseases like COVID-19, owing to its simple use and visual detection by fluorescence or color change (Ding et al., 2020). Broughton and group had come up with a CRISPR Cas12 based detection method which they claim to be the most rapid (<40) minute technique among isothermal nucleic acid based POC technique. This CRISPR Cas12-based technique is used to detect SARS-CoV-2 from extracted RNA samples of suspected patients (Chen et al., 2018; Chiu, 2018). This technique has been designed to perform simultaneous reverse transcription and isothermal amplification using RT-LAMP to the RNA extracted from nasopharyngeal swabs followed by Cas12-mediated detection of virus. The technique has been named DNA endonuclease targeted CRISPR trans reporter (DETECTR) assay (Broughton et al., 2020). DETECTR assay showed a comparable accuracy related to RT-PCR. Some key advantages of this assay are isothermal amplification thus avoiding the need of thermocycling, easy to use systems like lateral flow strips, avoiding the use of complex laboratory infrastructure. This technique can be easily mobilized to the hot spots of COVID-19 transmission to ease the diagnosis process. A distinct approach in response to the increasing demand of global rapid testing for detecting SARS-CoV-2 infection was showcased by Rauch et al. which is CRISPR Cas13 based diagnostic method. The test has been named as CREST (Cas13-based, Rugged, equitable, scalable testing) (Rauch et al., 2020). This method is sensitive and a field deployable procedure to combat diagnosis crisis in pandemic situations. The method is easy and can be used at minimal infrastructure sites. CREST uses easily available protein, low cost thermocyclers and easy to use fluorescent visualizers which makes it a very low cost and easily affordable diagnostic technique. An another portable, accurate and mobile phone based diagnostic assay was reported as an amplification free assay using Cas13a for directly detecting SARS-CoV-2. The assay utilizes patient nasal swab to detect SARS-CoV-2 infection which can be analyzed with the help of a smart phone, this technology has made testing portable and affordable in the low resource areas. It has exhibited limit of detection in 10 fM of RNA target. This technique is much advanced than CRISPR COVID (Hou et al., 2020) as previously mentioned in this manuscript, which provides qualitative results using isothermal amplification. The sensitivity of other CRISPR based tests have been taken into consideration while developing this assay. Using best combination of crRNAs for entire viral genome makes this assay best fit for point of care diagnosis (Fozouni et al., 2020). A highly accurate, single nucleotide variant detection system developed by IGIB India employs *Francisella novicida* (Fn-Cas9) based enzymatic readout for nucleotide detection and nucleobase identification. They have named this approach as FnCas9 Editor Linked Uniform Detection Assay (FELUDA) (Azhar et al., 2020).

## Protein-Based COVID-19 Diagnosis

**Figure 2:**
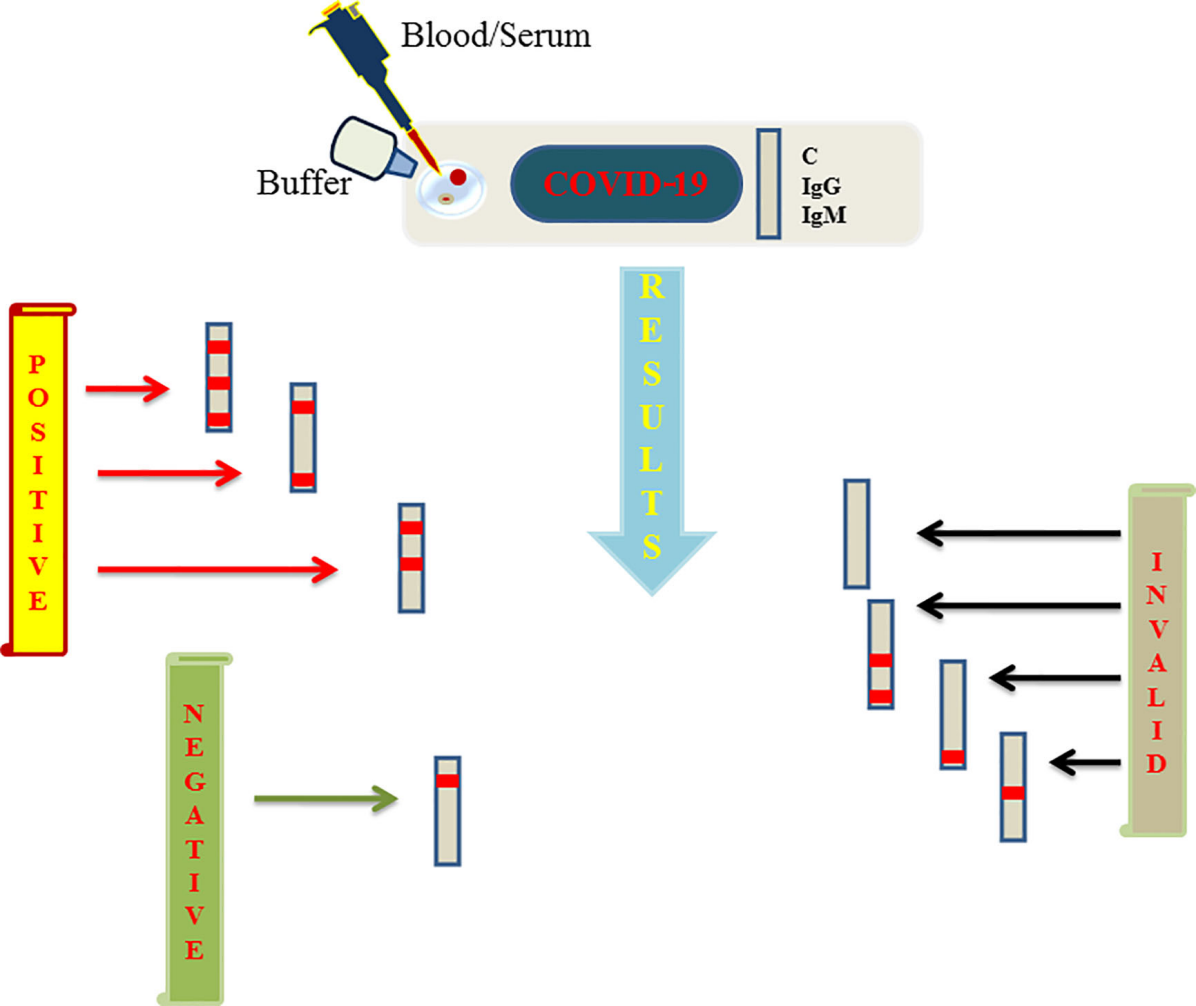
Figure represents schematics regarding the rapid and point of care test based on lateral flow assay (LFA) launched by various biotechnology companies and further approved by FDA to aid the COVID-19 testing. A sample well is flushed with blood/serum and buffer on the strip. Strip consists of three lines among which two are G and M detection lines representing IgG and IgM respectively and a quality control line represented as C line. If a red line appears on control, G and M or control and one among G/M, the test is said to be positive. Negative test shows only single redline on C line only. If C line is not visible the results are considered as invalid which needs to be repeated again.

Viral proteins as antigens or antibodies generated in response to viral infection can serve as the means of diagnosis in viral infectious diseases like COVID-19. Relying on viral proteins for detection is cumbersome as the viral load fluctuates during the course of infection, so antibodies can better serve the diagnosis process (To et al., 2020). It has been an age old practice to detect specific antigens by antibodies that are directed against these antigenic epitopes using immunoblot assays (Hartstein et al., 1989). COVID-19 can be diagnosed indirectly by detecting antibodies generated in the patient’s blood in a certain window period. However, there is a challenge of cross-reactivity between antibodies generated against SARS-CoV-2 and antibodies against other coronaviruses. A high frequency of cross reactivity was observed by a study carried out by Lv et al. where they had tested plasma samples taken from fifteen COVID-19 patients (Lv et al., 2020). According to a study carried out by Bin Ju et al. where the antibody response was characterized in eight COVID-19 patients and around 206 mAbs specific to SARS-CoV-2 receptor binding domain were also isolated. They observed the diverse antibody generation in the set of patients and proposed that such antibodies can serve as prophylactic and therapeutic strategies against COVID-19 (Ju et al., 2020). Liu et al. used SARS-CoV-2 IgG/IgM antibody test kit that was manufactured by a Chinese Biotechnology company and further approved by the China Food and Drug Administration. The protocol used around 5 ml of fasting blood of every participant. After collecting serum, the SARS-CoV-2 IgG/IgM were detected. The kit consists of three detection lines, control (C) line/zone, G zone and M zone. C line appears when the sample is flushed over it. A red test line in G and M zones will indicate the SARS-CoV-2 IgG/IgM in the samples. The test is to be repeated if the C line doesn’t appear on the strip (Liu et al., 2020). A schematic diagram showing the general protocol of IgG/IgM rapid and Point of Care COVID-19 diagnosis is depicted in **Figure 2**. Study conducted by Zhao et al. who had used enzyme linked immunosorbent assay (ELISA) kit developed by Baijing Wantai pharmaceutical company. The kit works on the principle of double antigen sandwich assay. A recombinant antigen containing receptor-binding domain (RBD) of the spike protein of SARS-CoV-2 expressed in mammalian cells was used as an immobilized horseradish peroxidase (HRP)-conjugate antigen. To detect IgM in the patient samples, IgM μ-chain capture method was used, however IgG was detected by using indirect ELISA kit based on recombinant nucleoproteins. They have claimed around 99% sensitivity of IgM and IgG antibodies for this assay (Zhao et al., 2020). Elevated levels of C reactive-protein and D-dimer and low levels of lymphocytes, blood platelets and leukocytes were shown by Guan et al. in the SARS-CoV-2 infected patients. The challenge in using them as biomarkers is that such markers are also found in different other ailments and abnormalities (Guan et al., 2020). Based on different techniques, there are various FDA approved diagnostic kits or kits with an emergency approval in the market. **Table 1** summarizes various such diagnostic kits used for diagnosis of COVID-19. A biosensor-based point of care test strategy was also reported by some research groups where they are claiming rapid antigen detection in saliva of COVID-19 positive patients. The strategy includes developing of a U bent Fiber optic probe over which the gold nanoparticles are immobilized. The anti-N-protein antibody (monoclonal) covalently conjugates with immobilized nanoparticles *via* thiol-PEG-NHS based binding. This biofunctionalized system are used to detect COVID-19 by applying saliva samples. The device is named as Fiber -Optic Biosensor device and works on the principle of monitoring an optical power loss in light (Murugan et al., 2020). Another setting of biosensor development for COVID-19 as highlighted by Pooja Nag et al. (2020) is an antibody immobilization on either polyaniline or gold nanoparticle coated optical fibers for specific detection of viral proteins in the sample, viral binding with the immobilized antibodies will change the refractive index in the local environment, thus causing change in the light intensity or absorption. Viral surface protein is also immobilized on the surface of optical fiber for the detection of IgG/IgM in the patient samples. The limit of detection claimed by the researchers in such setting was 100 U/ml in an hour (Nag et al., 2020). The sensitivity of such biosensor-based settings cannot be compared with RT-PCR sensitivity for a reason that antigen antibody interaction or immune response cannot be considered as precise indicators of pathogen infection or disease propagation. Another reason is that two types of pathogens can elicit same type of immune response which is a drawback of this setting in specificity.

**Table 1:** FDA approved diagnostic kits or kits with an emergency approval in the market to be used for the detection of COVID-19.

S/No	Name of testing kit	Technology used	Result time	Samples used	Authorization
01	InBios Smart Detect SARS-CoV-2 rRT-PCR Kit	RT-PCR	4 h	Nasal/Nasopharyngeal swabs	EUA
02	CO-DIAGNOSTICS INC. Logix Smart Coronavirus 2019 (COVID-19) Kit	RT-PCR	Not mentioned	Respiratory tract samples/serum	EUA
03	Gnomegen COVID-19 RT-Digital PCR Detection Kit	RT-digital PCR	Not mentioned	Not mentioned	EUA
04	ScienCell SARS-CoV-2 Coronavirus Detection Kit	RT-qPCR	Not mentioned	Respiratory specimen/serum	EUA
05	Luminex ARIES SARS-CoV-2 Assay	RT-PCR	2 h	Nasopharyngeal swabs	EUA
06	Luminex NxTAG CoV Extended Panel	RT-PCR	>2 h	Nasopharyngeal swabs	EUA
07	BD BioGX SARS-CoV-2 Reagents for the BD MAX™ System	RT-PCR	Not mentioned	Nasopharyngeal/ Oropharyngeal swabs	EUA
08	BD SARS-CoV-2 Reagents for the BD MAX™ System	RT-PCR	Not mentioned	Nasopharyngeal/ Oropharyngeal swabs	EUA
09	QIAGEN QIAstat-Dx Respiratory SARS-CoV-2 Panel	RT-PCR	1 h	Nasopharyngeal swabs	EUA
10	Abbot ID NOW™ COVID-19 Assay	Point of care/isothermal	15 min	Nasopharyngeal/ Oropharyngeal swabs	EUA
11	BGI Genomics Real-Time Fluorescent RT-PCR Kit	Taq Man RT-PCR	3 h	Throat swabs	EUA
12	Avellino CoV-2 Test	RT-PCR	1 h	Nasopharyngeal/ Oropharyngeal swabs	EUA
13	Avellino CoV-2 Test	PCR/point of care	30 min	Throat and nasal swabs	EUA
14	BioFire Diagnostics BioFire^®^ COVID-19 Test	Nested/Multiplexed/RT-PCR	1 h	Throat and nasal swabs	EUA
15	Primerdesign Ltd. COVID-19 genesig Assay	RT-PCR	<1 h	Nasopharyngeal/ Oropharyngeal swabs	EUA
16	ThermoFisher Scientific TaqPath COVID-19 Multiplex Diagnostic Solution	RT-PCR	<2 h	Nasopharyngeal/ Oropharyngeal swabs	EUA
17	The Centers for Disease Control and Prevention (CDC) 2019-nCoV Diagnostic Panel	RT-PCR	>2 h	Upper/lower respiratory specimen	EUA
18	Cellex qSARS-CoV-2 IgG/IgM Rapid Test	LFA	15-20 min	Blood/serum	EUA
19	TaqPath™ COVID-19 Combo Kit	RT-PCR	<1 h	Self-collected saliva	FDA

These approved kits are for the test of SARS-CoV-2 under Emergency Use Approval (EUA) and the information is available till April 9, 2020 which is mentioned here. It includes technique by which the testing kit operates, time of result and sample collection (https://hitconsultant.net/2020/04/23/in-depth-32-fda-approved-covid-19-testing-kits/#.XrZnkUQzbIV).

## Discussion

During the pandemic, the only way to tackle with the pathogen is to limit its spread which is only possible if the affected people get detected and separated at the earliest. This review has tried to compile the different diagnostic approaches used by academic labs and clinicians to diagnose COVID-19 disease since the identification of SARS-CoV-2 till now. Identification of SARS-CoV-2 in Wuhan, China using sequencing techniques was a major breakthrough (Rothan and Byrareddy, 2020) because its identification led scientists to progress for its diagnosis and therapeutic studies. The first recommended technique for its diagnosis by the CDC China was RT-PCR. This technique is much used during the current COVID-19 pandemic (Corman et al., 2020). It is the same technique that was used to diagnose SARS-CoV in 2002. The lessons learned from SARS-CoV outbreak has guided the very early identification of SARS-CoV-2 infection using sequencing and RT-PCR techniques. During the course of time, since its identification, there has been an immense study on developing rapid nucleic acid-based tests to detect COVID-19 disease among which SHERLOCK, CRISPR and other lateral flow based diagnostic kits are important to mention. These diagnostic approaches are parallelly competing in diagnostic accuracy with the RT-PCR-based diagnosis, however the biosensor related diagnosis needs more understanding and optimization to make them fit for pathogen diagnosis without the need of further confirmations using RT-PCR based -tests. Another approach was the establishment of serological-based diagnostic tests which are comparatively easy to handle and don’t need sophisticated machines or trained personnel like RT-PCR and can be easily used in the home settings so as to decrease exposure of health practitioners who are at high risk of encountering an infection during this pandemic. This manuscript has tried to bring in light various serological-based diagnostic approaches as depicted in **Figure 2** that is based on IgG/IgM antibodies. Asymptomatic spread of COVID-19 as reported by some research groups, made it crucial to develop multiplex and Point-of-Care techniques like isothermal amplification, CRISPR-based techniques and microfluidic techniques, so that they can be used to test the majority of the population and isolate infected persons mostly in remote areas, quarantine centers, in developing countries which lack enough resources and skills

Data on COVID-19 is evolving very rapidly, and there is no doubt that some of the specifics of this article may change as more studies become available. This review will help a reader to understand the established and other promising techniques in managing pandemic diseases like COVID-19.

## Author Contributions

JI and KUI conceived the idea. KUI wrote the review article. JI has read, corrected the write up, and finalized. All authors contributed to the article and approved the submitted version.

## Conflict of Interest

The authors declare that the research was conducted in the absence of any commercial or financial relationships that could be construed as a potential conflict of interest.
